# Biological Effects of F(ab′)2 Fragments Generated by Imlifidase From Anti‐HLA IgG Antibodies From Transplant Patients

**DOI:** 10.1111/tan.70502

**Published:** 2025-12-14

**Authors:** Magali Devriese, Ilaria Carelli, Lisa Giraldo, Alexis Pin, Noé Groshaeny, Sephora Da Silva, Robert Bockermann, Jean‐Luc Taupin

**Affiliations:** ^1^ Laboratoire d'Immunologie et Histocompatibilité, Hôpital Saint Louis Paris France; ^2^ INSERM U1342, Institut de Recherche Saint‐Louis Université Paris Cité Paris France; ^3^ Hansa Biopharma Lund Sweden

## Abstract

Imlifidase cleaves intact IgG molecules, generating F(ab′)2 fragments that still bind to the target antigen but are devoid of the Fc‐dependent effector functions, notably complement activation. Imlifidase (Idefirix) has been conditionally approved for desensitisation to enable kidney transplantation in highly sensitised adult patients. We hypothesised that the F(ab′)2 fragments can prevent the attachment of the full IgG at the time of anti‐HLA antibodies rebound when imlifidase action fades away, lowering the deleterious effect of the antibody burden on the transplant. This competition was explored in vitro using the Luminex Mixed or Single Antigen bead assays, and with an antibody detecting the specific imlifidase‐cleaved IgG. Functional assays for complement activation were also performed, which were the C1q bead assay and complement‐dependent cytotoxicity crossmatches. We selected sera from patients with a high amount of class I or class II anti‐HLA antibodies. Competition by imlifidase‐digested serum was observed in Luminex antibody‐binding assays and cellular complement activation assays. F(ab′)2 fragments generated from anti‐HLA antibodies after imlifidase cleavage had a biological impact on intact IgG binding, leading to reduced complement activation. Additional studies are needed to demonstrate whether these in vitro features have beneficial clinical consequences in vivo, that is, protecting the graft from potentially deleterious effects of DSA rebound.

## 1 Introduction

Highly sensitised patients awaiting organ transplantation have a substantially reduced likelihood of finding an HLA‐matched organ due to the presence of a wide array of anti‐HLA antibodies [1]. Identification of these antibodies is accomplished using the Luminex Single Antigen (LSA) bead assays, pre and post transplantation in the recipients' sera [2]. One approach to desensitisation involves the use of imlifidase, an immunoglobulin G (IgG)‐degrading enzyme derived from *Streptococcus pyogenes* [3, 4, 5]. This protease exhibits remarkable selectivity in cleaving human IgG at the lower hinge region, resulting in the generation of an intermediate transient compound called single‐cleaved IgG (scIgG), then in one F(ab′)2 and Fc fragments [6].

Both scIgG and F(ab′)2 are expected to maintain their ability to bind to their target antigens. However, scIgG exhibits only partial activity in terms of IgG effector functions, while F(ab′)2 fragments are known to lose their capacity to activate complement and Fc receptor‐mediated immune responses [7]. Notably, antibody‐mediated NK cell activation and Antibody‐Dependent Cell‐mediated Cytotoxicity coordinated by anti‐HLA antibodies are inhibited by imlifidase [8]. When imlifidase completes its role, it reduces the antibody burden on the transplant, leading to clinical improvement when used for treating ongoing humoral rejection or preventing rejection in recipients with donor‐specific antibodies (DSA) at the time of transplantation [9, 10].

Nevertheless, the impact of imlifidase might extend beyond simply cleaving IgG molecules and thereby separating immunoglobulin's antigen binding from effector functions. The transplanted organ might retain F(ab′)2 fragments on the surface of its cells, engaged with their specific HLA antigens. When IgG levels start to rise back due to de novo synthesis of new full IgG DSA molecules, a local competition could occur in the transplant. If the imlifidase‐cleaved and newly synthesised IgGs originate from the same B‐cell clones, their affinities for antigens will be identical. Consequently, the non‐cleaved IgG would hardly displace the F(ab′)2 fragments, potentially offering a protective effect to the organ for an unknown period.

To explore this potential benefit of imlifidase, we applied complementary approaches relying on Luminex bead assays and cellular crossmatches to mimic the transplant, using sera treated in vitro with imlifidase.

## 2 Materials and Methods

### 2.1 Selection of Patients' Sera

Sera were selected from our local database of MFI values from 225,133 LSA assays performed for the patients' routine follow‐up between 15th September 2015 and 1st July 2023 at the Histocompatibility Laboratory of the Saint‐Louis Hospital in Paris, France. 15th September 2015 represents the day when systematic EDTA pre‐treatment of serum was implemented, in order to circumvent complement interference [11]. Selection criteria were saturated Mean Fluorescence Intensity (MFI) values over 20,000 in pure and 1:10 diluted serum, and LSA profiles presenting no more than two antibodies per locus targeting two antibody‐verified eplets according to HLA Eplet Registry [12].

### 2.2 Imlifidase In Vitro Cleavage of Serum IgG

Imlifidase was provided at 10 mg/mL by Hansa Biopharma (Lund, Sweden), and stored frozen at −80°C until use, in aliquots for single use. Imlifidase was diluted in serum to 25 μg/mL and incubated at 37°C for 2 h. Aliquots of the treated sera were named after ‘Sxi’, with x and i representing the serum number and imlifidase respectively, frozen and used once avoiding freeze–thaw cycles. A negative control serum (named after ‘N’), pooled from AB group non‐allosensitised blood donors, was used and treated with imlifidase in identical conditions, and named after ‘Ni’.

### 2.3 SDS‐PAGE Electrophoresis and Fc‐Specific Western Blot

SDS‐PAGE electrophoresis and western‐blot analysis were carried out in order to demonstrate the efficiency of the cleavage and the distribution of the full, scIgG and F(ab′)2 fragments of serum IgG. Experimental conditions are reported in the legend of Figure S2.

### 2.4 Luminex Bead Assays

The LABScreen Mixed (LSM12 Lot 24, One Lambda, West Hills, California) and the LABScreen Single Antigen (LSA) HLA Class I and Class II (LS1A04 Lot 14 and LS2A01 Lot 15, One Lambda) were used according to the manufacturer's instructions. The LABScreen Mixed kit is a screening test for which each bead is coated with a wide variety of class I or class II antigens to enable broad detection of anti‐HLA antibodies. In contrast, the LSA beads are coated with a unique antigen per bead type.

Raw MFI data acquired on a LABScan 200 apparatus (Luminex, Austin, Texas), were normalised for each bead by subtracting the MFI of the HLA‐negative control bead from the same serum. All sera were pre‐treated for 10 min at room temperature with EDTA at a final concentration of 0.01 M.

LSA assays were performed with three conjugates: (1) the recommended Phycoerythrin (PE)‐conjugated goat anti‐human IgG (LS‐AB2, One Lambda) recognising the Fc fragment, that is, the full IgG and the scIgG; (2) a biotinylated goat anti‐human Fab region (kappa+lambda light chains) recognising the F(ab′)2 fragments, that is, from full IgG, scIgG and digested F(ab′)2 fragments (#2085‐08, SouthernBiotech, Birmingham, Alabama), followed by a PE‐conjugated streptavidin (One Lambda); and (3) a mouse anti‐imlifidase‐cleaved hinge of IgG antibody, specifically recognising a neo‐epitope generated by the enzyme cleavage at the C‐terminus of the imlifidase‐digested F(ab′)2 fragments (Ab01182‐1.1, clone C2095, Absolute Biotech, Boston, Massachusetts), followed by a PE‐conjugated anti‐mouse IgG (#405307, Biolegend, San Diego, California) [13]. These three conjugates were hereafter named ‘Fc’, ‘Fab’ and ‘Hinge’ respectively. Hinge and Fc were used at 1:100 dilution, and Fab at 1:400 (titration data not shown). Of note, the Hinge antibody does not react with IgG2 [13], but this isotype is very rarely accompanying anti‐HLA antibodies of IgG1 and/or IgG3 isotypes.

The C1q LSA assay (C1qScreen, One Lambda) was performed according to manufacturer's instructions. Briefly, 5 μL of serum was incubated with LSA beads and with 5 μL of recombinant C1q for 20 min at room temperature. A PE‐conjugated monoclonal anti‐human C1q antibody was added and incubated for 20 min. Then beads were washed and resuspended in 70 μL of Phosphate Buffered Saline (PBS), before data acquisition. All experiments were conducted at a controlled ambient temperature between 20°C and 25°C.

For competition assays, Mixed or LSA beads were first incubated 30 min with Sxi or Ni. After 5 washes, the beads were incubated with PBS for an additional 10 min, then washed again 5 times to remove any traces of imlifidase. Beads were subsequently incubated with non‐treated serum Sx and then, Fc, Fab, Hinge and C1q binding were analysed. MFI loss after Fc staining was calculated in percentage as (MFISxi+Sx − MFINi+Sx)/MFINi+Sx * 100.

### 2.5 Complement‐Dependent Cytotoxicity Crossmatches

The complement‐dependent cytotoxicity (CDC) crossmatches were performed on T and B lymphocytes extracted from lymph node or spleen of organ deceased donors. HLA typing of the 11 loci (A, B, C, DRB1, DRB3/4/5, DQA1, DQB1, DPA1, DPB1) was performed with LinkSeq SureTyper SSP PCR assay (One Lambda). T and B lymphocytes were isolated with magnetic beads by negative separation (EasySep Isolation Kit, #89671 and #89684, Stemcell Technologies, Vancouver, Canada) and used for class I or class II sera respectively. Each serum was tested with 3 different donor cells. Briefly, sera were pre incubated at 56°C for 30 min to inhibit the complement system. Thereafter, sera were mixed with dithiothreitol (DTT) at 15 mM during 10 min at room temperature in 50% v/v, to inactivate human complement and IgM. In a Terasaki plate, 1 μL of donor T or B lymphocytes per well were incubated with 2 μL of these pre‐treated sera at 22°C for 30 min. After a washing step with 6 μL of PBS per well, high speed centrifugation during 15 s and flicking, 3 μL per well of rabbit serum as complement source (ABC Complement, CABC‐5, One Lambda) was added at 22°C for 1 h. Directly after, 3 μL per well of a fluorescent reagent staining dead cells orange and live cells green (Fluoroquench AO/EB, FQAE500, ThermoFisher Scientific, Waltham, Massachusetts) were added and incubated 3 min before reading the plate on a fluorescence microscope. A cell lysis score was assigned to each well according to the NIH (National Institutes of Health) cytotoxicity score, from score 1 representing < 10% dead cells, score 2, 4, 6 and 8 for 10%–20%, 20%–50%, 50%–80% and > 80% dead cells respectively. The positivity threshold for CDC crossmatches is set at score 2. Each well and condition was performed in duplicate, and results were expressed as average of duplicates.

For competition in CDC crossmatches, 4 scenarios were performed, called (1) N+Sxi, (2) N+Sx, (3) Ni+Sx and (4) Sxi+Sx, as follows: donor T or B lymphocytes were first incubated with N in scenarios (1) and (2), with Ni in scenario (3) or Sxi in scenario (4), in 50% v/v for 30 min on ice. Cells were washed with 1 mL of PBS and centrifuged 10 min. Supernatant was discarded and cells were incubated with the imlifidase‐treated serum Sxi in scenario (1) or the non‐treated serum Sx in scenarios (2), (3) and (4), for 30 min at 22°C. Then complement was added, and after incubation followed by fluorescent staining, cytotoxicity was evaluated.

### 2.6 Statistical Analysis

Statistical analysis and graphs were performed using GraphPad software version 8.0 (San Diego, California). Unpaired‐*t* tests were used for statistical analysis.

## 3 Results

### 3.1 Cleavage of Serum IgG by Imlifidase

We selected sera with very high MFI values in order to saturate the LSA beads antigens as much as possible and thus be able to observe competition in the bead‐based assays. Thus, S1, S2 and S3 were selected for HLA class I, and S4, S5, S6 and S7 for HLA class II (Table 1).

**TABLE 1:** Characteristics of the 7 patients' sera studied.

Sample	S1		S2	S3	S4	S5	S6	S7	
Locus	A	B	A	A	DQB	DQB	DQA, DQB	DQB	DPB
Eplet	82LR, 95V		127K	79GT	84QL	84QL	40GR, 76V, 55PP	52PQ	84DEAV
Number of beads	9	19	9	26	20	20	16	8	20
Positive LSA beads	A*02:01	B*13:01	A*02:01	A*01:01	DQA1*02:01DQB1*02:01	DQA1*02:01DQB1*02:01	DQA1*03:01DQB1*02:01	DQA1*01:01DQB1*05:01	DPA1*01:03DPB1*01:01
	A*02:03	B*13:02	A*02:03	A*02:01	DQA1*03:01DQB1*02:01	DQA1*03:01DQB1*02:01	DQA1*04:01DQB1*02:01	DQA1*01:02DQB1*05:02	DPA1*02:01DPB1*01:01
	A*02:06	B*27:05	A*02:06	A*02:03	DQA1*04:01DQB1*02:01	DQA1*04:01DQB1*02:01	DQA1*05:01DQB1*02:01	DQA1*01:03DQB1*06:01	DPA1*02:01DPB1*05:01
	A*23:01	B*15:13	A*23:01	A*02:06	DQA1*05:01DQB1*02:01	DQA1*05:01DQB1*02:01	DQA1*03:03DQB1*04:01	DQA1*01:01DQB1*06:02	DPA1*02:02DPB1*05:01
	A*24:02	B*15:16	A*24:02	A*03:01	DQA1*02:01DQB1*02:02	DQA1*02:01DQB1*02:02	DQA1*04:01DQB1*04:02	DQA1*01:02DQB1*06:02	DPA1*01:03DPB1*03:01
	A*24:03	B*37:01	A*24:03	A*11:01	DQA1*02:01DQB1*04:01	DQA1*02:01DQB1*04:01	DQA1*02:01DQB1*03:01	DQA1*01:03DQB1*06:03	DPA1*01:05DPB1*03:01
	A*25:01	B*38:01	A*68:01	A*11:02	DQA1*03:03DQB1*04:01	DQA1*03:03DQB1*04:01	DQA1*03:01DQB1*03:01	DQA1*01:02DQB1*06:04	DPA1*02:01DPB1*03:01
	A*32:01	B*44:02	A*68:02	A*26:01	DQA1*02:01DQB1*04:02	DQA1*02:01DQB1*04:02	DQA1*05:03DQB1*03:01	DQA1*01:02DQB1*06:09	DPA1*01:03DPB1*06:01
	A*69:01	B*44:03	A*69:01	A*29:01	DQA1*04:01DQB1*04:02	DQA1*04:01DQB1*04:02	DQA1*06:01DQB1*03:01		DPA1*02:01DPB1*06:01
		B*49:01		A*29:02	DQA1*02:01DQB1*03:01	DQA1*02:01DQB1*03:01	DQA1*05:05DQB1*03:19		DPA1*02:01DPB1*09:01
		B*47:01		A*30:01	DQA1*03:01DQB1*03:01	DQA1*03:01DQB1*03:01	DQA1*02:01DQB1*03:02		DPA1*02:02DPB1*10:01
		B*51:01		A*30:02	DQA1*05:03DQB1*03:01	DQA1*05:03DQB1*03:01	DQA1*03:01DQB1*03:02		DPA1*01:03DPB1*11:01
		B*51:02		A*31:01	DQA1*06:01DQB1*03:01	DQA1*06:01DQB1*03:01	DQA1*03:02DQB1*03:02		DPA1*02:02DPB1*11:01
		B*52:01		A*33:01	DQA1*05:05DQB1*03:19	DQA1*05:05DQB1*03:19	DQA1*02:01DQB1*03:03		DPA1*02:01DPB1*13:01
		B*53:01		A*33:03	DQA1*02:01DQB1*03:02	DQA1*02:01DQB1*03:02	DQA1*03:01DQB1*03:03		DPA1*02:02DPB1*13:01
		B*57:01		A*34:01	DQA1*03:01DQB1*03:02	DQA1*03:01DQB1*03:02	DQA1*03:02DQB1*03:03		DPA1*03:01DPB1*13:01
		B*57:03		A*34:02	DQA1*03:02DQB1*03:02	DQA1*03:02DQB1*03:02			DPA1*02:01DPB1*14:01
		B*58:01		A*36:01	DQA1*02:01DQB1*03:03	DQA1*02:01DQB1*03:03			DPA1*02:01DPB1*17:01
		B*59:01		A*43:01	DQA1*03:01DQB1*03:03	DQA1*03:01DQB1*03:03			DPA1*01:03DPB1*19:01
				A*66:01	DQA1*03:02DQB1*03:03	DQA1*03:02DQB1*03:03			DPA1*03:01DPB1*20:01
				A*66:02					
				A*68:01					
				A*68:02					
				A*69:01					
				A*74:01					
				A*80:01					

Abbreviation: LSA, Luminex Single Antigen.

**FIGURE 1:**
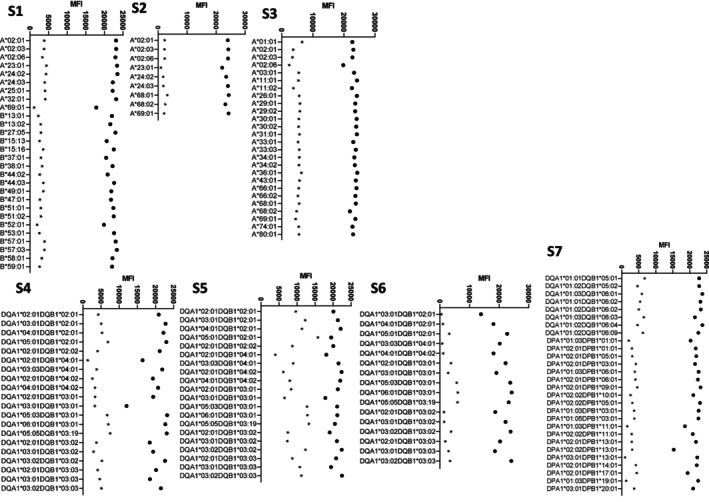
Luminex Single Antigen profiles of the 7 studied sera. Distribution of the MFI values of sera S1 to S7 before in vitro treatment with imlifidase (full circle) and after treatment (stars). Antigens of the positive LSA beads are on the vertical *X* axis.

S1 to S7 were treated in vitro with imlifidase in conditions supposed to fully cleave anti‐HLA IgG. In LSA assay, MFI values decreased with Fc staining for all digested sera Sxi, from (average MFI ± standard deviation) 21,854 ± 1637 to 4505 ± 1456 (Figure 1, Figure S1A). Average MFI losses ranged from −53% to −91%, reaching an average of −78% ± 7% (Figure S1B). S5 showed the weakest cleavage with average MFI decreasing from 207,08 ± 1371 to 9820 ± 2919 and average MFI loss at −53%.

Conversely, MFI values drastically increased with Hinge staining, from 88 ± 35 to 22,227 ± 1992, confirming the presence of imlifidase‐cleaved IgG (Figure S1C).

SDS‐PAGE analysis confirmed that serum IgG was successfully cleaved by imlifidase in the 7 sera (Figure S2A,B). In addition, no intact IgG or scIgG could be detected using Fc‐specific Western analysis (Figure S2C,D).

### 3.2 F(ab′)2 Competition With Intact IgG in Bead‐Based Assays

Once serum IgG was efficiently cleaved by imlifidase, competition assays were attempted, first using the Mixed beads. This kit contained 12 class I and 5 class II beads each expressing a mixture of several HLA antigens. Beads were first incubated with the imlifidase‐treated serum Sxi, or treated negative control serum Ni. Then serum was removed by extensive washing and beads were incubated with non‐treated serum Sx. In case beads would be saturated enough by the F(ab′)2 fragments, the Fc‐specific conjugate would detect less or even no binding of intact IgG from Sx serum. Replacing Sxi by Ni would not impair at all the binding of Sx.

**FIGURE 2:**
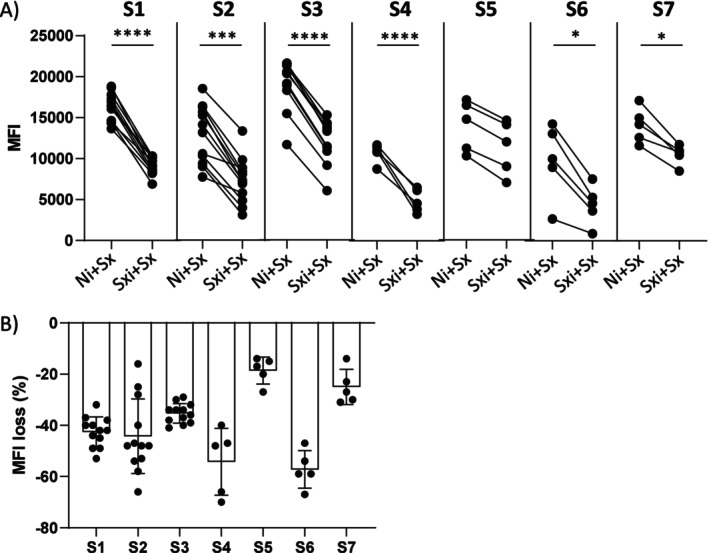
Competition assays with Luminex Mixed beads. Panel (A) Luminex Mixed beads were first incubated with the indicated imlifidase‐treated serum Sxi or treated negative control serum Ni. After washes, the same non‐treated serum Sx was added, then detected with the anti‐Fc conjugate. Unpaired‐*t*‐tests were performed for each serum tested, presented in the graphs by asterisks as **** for *p* < 0.0001, ** for *p* < 0.01 and * for *p* < 0.05. Panel (B) bar chart presenting percentages of MFI loss in the competition condition Sxi+Sx compared with the Ni+Sx condition, for each serum studied.

A significant decrease of binding was observed in the competition condition Sxi+Sx compared to Ni+Sx for the 6 patients' sera S1, S2, S3, S4, S6 and S7 (*p* < 0.0001, *p* = 0.0002, *p* < 0.0001, *p* < 0.0001, *p* = 0.05 and *p* = 0.01 respectively, Figure 2A) with average MFI losses of −43%, −44%, −35%, −54%, −57% and −25% respectively (Figure 2B). In contrast, for S5, MFI did not statistically significantly decrease (*p* = 0.2, Figure 2A) with average MFI losses at −19% (Figure 2B).

**FIGURE 3:**
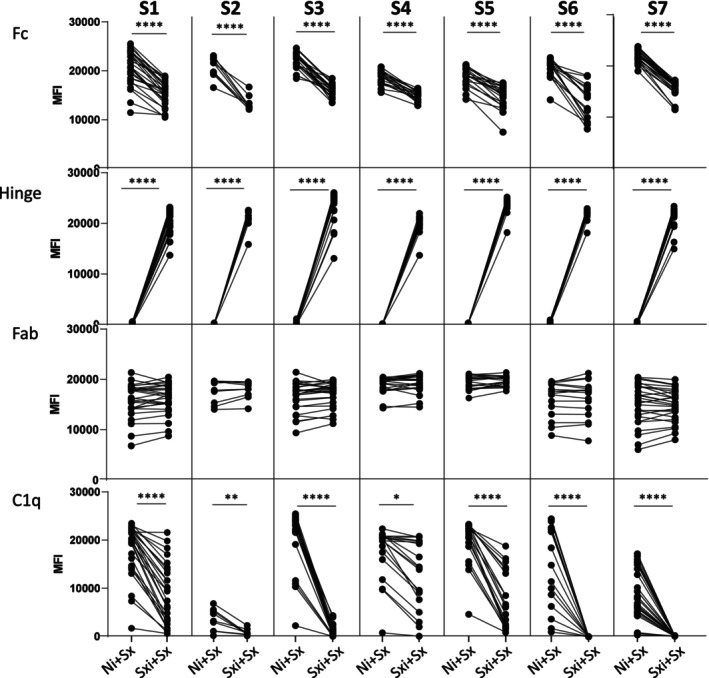
Competition assays with Luminex Single antigen beads. Luminex Single antigen beads were first incubated with imlifidase‐digested sera Sxi or digested negative control serum Ni before adding the non‐treated serum Sx, then the anti‐Fc, anti‐Hinge, anti‐Fab or anti‐C1q detection conjugates. Unpaired‐*t*‐tests were performed for each serum tested, presented in the graphs by asterisks as **** for *p* < 0.0001, *** for *p* < 0.001, ** for *p* < 0.01 and * for *p* < 0.05.

**FIGURE 4:**
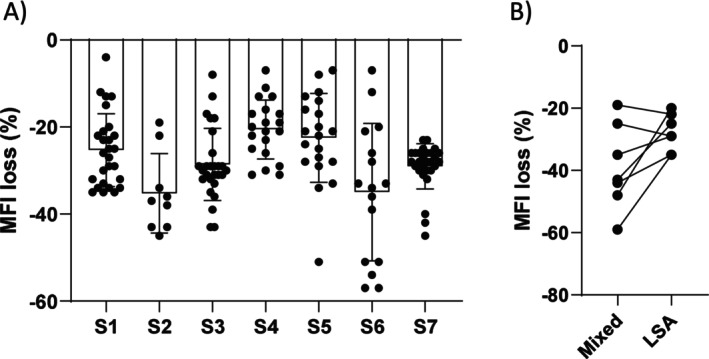
MFI loss in Luminex Single antigen competition assay. Panel (A) bar chart presenting percentages of MFI loss in the competition condition Sxi+Sx compared with the Ni+Sx condition, for each serum studied in LSA assays with anti‐Fc conjugate. Panel (B) comparison between average percentage of MFI loss for each serum in competition condition with mixed beads and LSA.

Thereafter, we performed the same protocol with LSA beads and utilising Fc, Hinge and Fab conjugates (Figure 3). With the Fc staining, MFI values decreased significantly in the competition condition for the 7 sera (*p* < 0.0001) as average MFI values in Ni+Sx and Sxi+Sx conditions were 20,125 ± 1957 and 14,398 ± 1961 all sera combined. All average MFI losses were below −20% with a mean for all sera at −28% ± 9% (Figure 4A). Average MFI losses bper serum, between Mixed and LSA assays were not statistically significant (*p* = 0.07) (Figure 4B).

However, blocking was not complete as Fc showed residual staining, evidencing either partial displacement of F(ab′)2 fragments or the presence of free HLA epitopes not occupied by the F(ab′)2 fragments. The Hinge conjugate highlighted the presence of imlifidase‐digested IgG on the beads for all sera, as MFI values were significantly increased in the Sxi+Sx condition (*p* < 0.0001 for S1 to S7; Figure 3). As expected, MFI values with Fab were not significantly different between Ni+Sx and Sxi+Sx conditions for all sera (*p* = 0.7 for S1 and S2, *p* = 0.6 for S3 and S4, *p* = 0.5 for S5, *p* = 0.9 for S6 and *p* = 0.8 for S7, Figure 3).

### 3.3 F(ab′)2 Competition With Intact IgG for Complement C1q Binding

Based on the observation that F(ab′)2 fragments competed with intact IgG on LSA beads, suggesting possible interference with IgG‐mediated complement activation, we examined their potential to inhibit C1q binding in competition LSA assays. Under Sxi+Sx conditions, we observed a significant decrease of C1q binding for all sera tested (*p* < 0.0001 for S1, S3, S5, S6 and S7; *p* = 0.005 for S2; *p* = 0.03 for S4; Figure 3). This result suggested that the intact IgG at the same concentration in serum as its imlifidase‐generated F(ab′)2 fragments, may not be able to displace the F(ab′)2 fragments pre‐adsorbed on the beads.

### 3.4 F(ab′)2 Competition With Intact IgG in CDC Crossmatches

CDC crossmatches were performed to explore if the F(ab′)2 competition could also reduce cell cytotoxicity. Experiments were performed for each of the 7 sera with lymphocytes from 3 different donors. Characteristics of cells were described in Table 2, especially their HLA typing as it was dependent on the antigens recognised by each serum tested.

**TABLE 2:** Characteristics of the sera and organ deceased donor cells used for the CDC crossmatches.

Sample	Locus	(Symbol in Figure 5) HLA typing of donor cells	Donor antigens targeted by serum	
			Number	Antigens
S1	A, B	(●) A24 B35	2	A24
		(■) A3 A24 B18 B44	2	A24 B44
		(▲) A2 A11 B13 B35	2	A2 B13
S2	A	(●) A24	2	A24
		(■) A3 A24	1	A24
		(▲) A2 A11	1	A2
S3	A	(●) A3 A11	2	A3 A11
		(■) A11 A33	2	A11 A33
		(▲) A30 A68	2	A30 A68
S4	DQB	(●) DQ7	2	DQ7
		(■) DQ7	2	DQ7
		(▲) DQ7 DQ6	1	DQ7
S5	DQB	(●) DQ7	2	DQ7
		(■) DQ7	2	DQ7
		(▲) DQ6 DQ9	1	DQ9
S6	DQA, DQB	(●) DQA1*05 DQ7	4	DQA1*05 DQ7
		(■) DQA1*03 DQA1*05 DQ7	4	DQA1*03 DQA1*05 DQ7
		(▲) DQA1*01 DQA1*05 DQ7 DQ6	2	DQA1*05 DQ7
S7	DQB, DPB	(●) DQ6 DQ7 DP4	1	DQ6
		(■) DQ4 DQ6 DP4 DP23	1	DQ6
		(▲) DQ6 DP4 DP13	3	DQ6 DP13

In addition to the Sxi+Sx competition condition, three control scenarios were performed: (1) N+Sxi as the negative control condition, for which the imlifidase‐cleaved serum should display no cytotoxicity; (2) N+Sx as the positive control condition, for which the full IgG DSA should kill the cells; (3) Ni+Sx as a control for the complete removal of imlifidase at the flicking step after the first incubation of the enzyme‐treated serum with the cells, which should also kill the cells.

**FIGURE 5:**
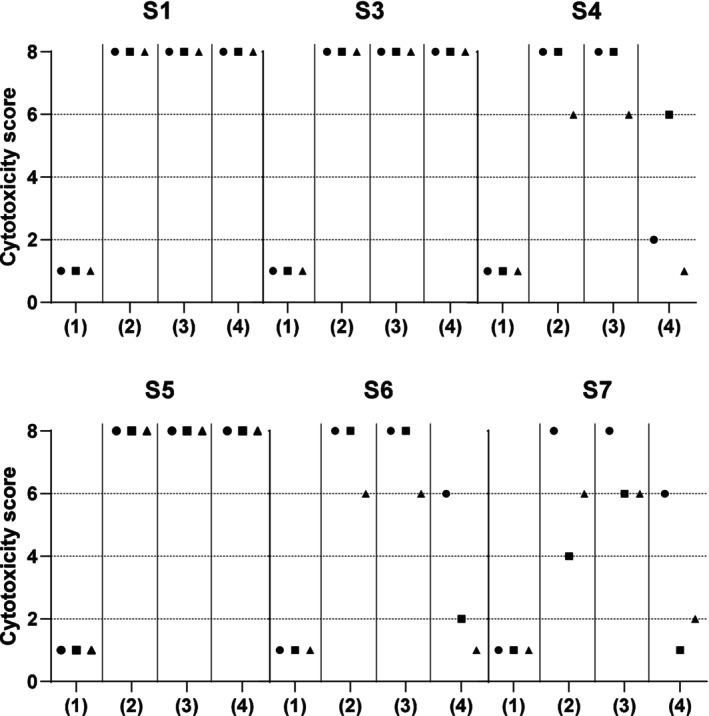
Competition in CDC crossmatch assays. The indicated serum (S1 and S3 for class I, S4 to S7 for class II) was tested against T or B lymphocytes from three different donors (indicated with a circle, a square and a triangle), according to antibody/typing associations reported in Table 2. Results are presented as NIH cytotoxicity scores from 1 (negative) to 8 (> 80% dead cells). Four conditions were tested for each serum/cell combination: (1) N+Sxi, (2) N+Sx, (3) Ni+Sx and (4) Sxi+Sx.

The results were different depending on the sera. Among the 3 class I‐specific sera, no decrease of S1 and S3 cytotoxic potential (at the maximal score 8) was observed in the competition condition (Figure 5). Regarding S2, despite its very high antibody level, it did not show any cytotoxicity in the control N+Sx and Ni+Sx conditions, therefore, competition could not be evaluated (result not shown).

Among the 4 class II‐specific sera, competition was observed for S4, S6 and S7 to various extent, from the initial score 4–8 down to score 1–6 depending on the serum/cell combination, but always with a lower score in the competition condition than in the N+Sx and N+Sxi control conditions (Figure 5). However, for serum S5, no competition was detected (Figure 5).

## 4 Discussion

This study aimed to assess the biological effects of anti‐HLA antibodies from highly sensitised patients, after the action of imlifidase. Through binding assays on HLA beads and cellular CDC crossmatches, we could evidence a competition between the F(ab′)2 fragments generated by imlifidase and intact IgG of the same epitope specificity. Overall, these results suggest that in vivo, imlifidase‐generated F(ab′)2 could act as decoys shielding the graft from the binding of residual intact DSA.

This protective effect would only occur in vivo if the F(ab′)2 fragments bind and persist long enough on the donor organ. However, the turnover rate of an HLA molecule bound to a F(ab′)2 fragment is unknown. Rapid turnover could result in the quick internalisation of F(ab′)2 and replacement by new HLA molecules, becoming free targets available for intact DSA. Therefore, a too fast turnover would reduce the protective effect of F(ab′)2. Nevertheless, imlifidase is used for highly sensitised patients with limited transplant options, and high anti‐HLA antibody levels suggest continuous de novo synthesis, giving imlifidase substrate for its proteolytic action over its full period of activity. Thus, F(ab′)2 fragments may accumulate with time and provide protection beyond the initial hours, potentially lasting several days if F(ab′)2‐HLA complexes persist on graft cells. Overall, several hypotheses are raised here, that were outside of the scope of the present article. Nonetheless we could demonstrate in vitro a certain protective effect of imlifidase generated F(ab′)2 fragments. This warrants the exploration of the in vivo situation, for a better understanding of the pharmacodynamics and downstream effects of imlifidase.

Our results show that the competition caused by pre‐existing F(ab′)2 fragments towards intact IgG added in a second step, can be detected with bead‐based assays for IgG and C1q binding detection, and with a cellular CDC crossmatch assay. However, our results show that the strength of the competition varies depending on the sera used for the bead‐based assays, and the sera/cell combinations for the CDC crossmatches. Regarding the bead‐based assays, the detected competition was far from complete, despite the fact that very strong sera were chosen, that contained antibodies at saturating MFI and capable of C1q binding. In addition, we used for competition the exact same serum samples, as might occur in vivo where patients F(ab′)2 fragments should face the same paratopes embedded into a full IgG molecule when DSA rebound occurs. For S5, a significant competition occurred in LSA assay compared to the Mix beads, even though a decrease is observed.

Partial binding of intact IgG could mean that intact IgG has displaced only a fraction of the F(ab′)2 decoy fragments on the beads, despite the fact that intact IgG and F(ab′)2 have the same avidity for the antigen. In addition to displacement, simple desorption could theoretically occur, but anti‐HLA IgG binding to LSA beads is extremely stable, with no detectable loss of bead MFI after 10 days of storage at 4°C (results not shown). Finally, the HLA antigen density on the surface of beads, even if unknown, is significantly higher than serum can saturate, leaving epitope sites free to bind intact IgG at the second incubation.

Due to the saturation of the Luminex photomultiplier (at approximately 20,000 MFI), antibodies from the second incubation may not be detected. However, our experimental scheme makes it possible to differentiate between the Fc fragment on intact IgG and the F(ab′)2 generated by imlifidase. In fact, staining with the anti‐F(ab′)2 fragment‐specific conjugate (Fab) does not impact MFI values between the first and second incubations. Using lower concentrations of the F(ab′)2 fragment in the first step would increase the number of free epitope sites for the second step, thereby allowing detection of additional anti‐HLA antibody binding but concomitantly reducing the chance of detecting competition.

To avoid the limitation of a too high epitope density on LSA beads for clearly detecting competition, we explored the use of screening Luminex Mixed Assay beads. The Mixed beads express a variety of HLA antigens on any given bead, thereby lowering the relative amount and density of each antigen. However, this does not always mean that the density of target eplets and epitopes decreases as they may be shared among different antigens, meaning that distinct antigens may bind serum antibodies to a similar degree. Therefore, when using sera with extremely high MFI values for many different antigens in the LSA assay, the risk that these sera contain many antibodies of different eplet specificities is high, making competition similarly difficult to detect with Mixed and Single antigen beads.

We observed that the C1q assay exacerbated the competition observed with IgG detection assays, thus supporting the initial hypothesis. Indeed, the C1q assay requires, in order to being positive, that the density of Fc fragments be sufficient to bind C1q hexamers with high enough avidity to generate a stable interaction. By randomly occupying a large number of epitope sites, the F(ab′)2 fragments widen the distance between the remaining free sites available to the added intact IgG, thus considerably limiting the ability to efficiently bind C1q. Consequently, the C1q bead assay confirms the results obtained with the IgG assays.

The C1q assay also had the advantage of demonstrating a biological function for this competition, showing altered complement‐dependent cytotoxicity, just as CDC crossmatches do. Despite CDC crossmatch correlating with high DSA MFI values, serum S2 proved non‐cytotoxic. Nevertheless, it efficiently bound C1q suggesting that the IgG isotypes of its antibodies were complement activating, although we did not perform IgG subclass determination. No competition in CDC crossmatch could be evidenced with the other two class I sera, whereas it was detectable with 3 out of the 4 class II sera. Again, antigen density may explain this. As class I antigens are highly expressed on T and B cells, DSA saturation is probably not necessary in order to kill cells and competition may therefore be difficult to demonstrate. All sera we selected for class II positivity targeted DQ antigens (and DP for S7), known to be much less expressed than DR [14]. Therefore, saturation may be easier to achieve with the F(ab′)2 fragments and competition more easily detectable. Furthermore, the turnover rate of class II molecules on B lymphocytes could be much slower than that of class I, leaving class II occupied for much longer, facilitating detection of competition. As crossmatches are performed at 22°C, both mechanisms may coexist.

Overall, sera tested show large heterogeneity in biological effector functions, as it has already been reported [15]. As said above, MFI saturation on beads does not allow predicting the true strength or level of DSA in a serum, but can be improved by a serum dilution assay [16]. The oligoclonal or polyclonal status of a serum will interfere by involving only one or many antigens on a bead or cell, depending on the variety of private or public recognised epitopes. In CDC assays the target cells come from distinct donors and may not behave identically, even though they shared the same target antigens [15]. The efficiency of competition can also vary depending on the epitope/antigen/locus studied, leading us to examine 7 sera targeting different eplets on different class I and II antigens and loci. These antibodies recognised public mono‐epitope or bi‐epitope present on several HLA antigens with an impact on a large number of beads, probably mimicking what might occur against a donor.

Overall, in vitro LSA and cellular approaches demonstrated that imlifidase‐generated F(ab′)2 fragments remained functional in antigen binding, offering the possibility of inhibiting binding to intact IgG and, consequently, reducing Fc effector functions. This F(ab′)2 decoy phenomenon should protect the graft against DSA loading, or at least be harmless if too low or even absent.

## Author Contributions

M.D., I.C., A.P., N.G., S.D.S., R.B. and L.G. performed the research. M.D., I.C., R.B. and J.‐L.T. analysed the data. M.D., R.B. and J.‐L.T. designed the research study and wrote the paper.

## Funding

This work was supported by public funding agencies (ANR EPIHLA AAPG2022 to J.‐L.T.), the French Agence de la Biomédecine (AOR 2022), non‐profit organisations (Fondation pour la Recherche Médicale 2022 to M.D.) and a research grant from Hansa Biopharma.

## Ethics Statement

Patient's sera used for this study were leftovers from blood drawings performed for follow‐up of their anti‐HLA antibody profile, from patients who gave their informed consent to their use for research purposes in transplantation.

## Conflicts of Interest

R.B. is employed by Hansa Biopharma and is the holder of shares in Hansa Biopharma AB. The other authors declare no conflicts of interest.

## Supporting information


**Figure S1:** IgG cleavage by imlifidase in the sera studied. Imlifidase‐treated sera S1i to S7i and non‐treated sera S1 to S7 were tested in LSA assay with an anti‐Fc (Panel A) or an anti‐Hinge conjugate (Panel C). Unpaired‐*t*‐tests were performed for each serum tested, presented in the graphs by asterisks as **** for *p* < 0.0001. Bar chart of percentages MFI loss in digested sera were depicted in panel B.

**Figure S2:** SDS‐PAGE and Western Blot analysis of imlifidase cleavage. SDS‐PAGE (Panels A and B) and Fc‐specific Western Blot (Panels C and D) were performed on non‐treated sera S1 to S7 and imlifidase‐treated sera S1i to S7i to evaluate the efficacy of IgG cleavage by imlifidase. STD = molecular size stand. Experiment was performed as follows. For size separation, the serum samples were loaded onto 10‐well 4%–20% Mini PROTEAN TGX SDS‐PAGE (Biorad, Hercules, California) and run at 200 V for 40 min. Gels were activated for 2 min and picture acquired by ChemiDoc MP system (Biorad) using Image Lab5.2. For Fc‐specific Western Blot analysis, gels were shortly rinsed in water and blotted onto nitrocellulose using Mini Trans‐Blot Turbo Transfer Packs (#1704158, Biorad) 2.4 A on 25 V for 7 min. Membranes were blocked in 5% Skim milk powder for 60 min. Membranes were incubated with Biotin‐SP F(ab′)2 Fragment Goat Anti‐Human IgG Fcg‐specific (#109‐066‐098, Jackson ImmunoResearch, West Grove, Pennsylvania). Streptavidin‐AF647 diluted in Tris Buffered Saline‐Tween was used for detection on ChemiDoc MP (Biorad) with suitable filter. For S2i, S3i and S6i, serum sample preparations even under non‐reducing conditions, seems to show reducing activity in SDS‐PAGE assays, showing itself as 45 kDa Fab′ bands in the imlifidase cleaved samples. The 100 kDa band in the S4 sample is probably caused by a different serum protein than F(ab′)2. This might be due to treatment differences or disease status, for example, acute phase proteins in the serum.

## Data Availability

The data that support the findings of this study are available from the corresponding author upon reasonable request.

## References

[1] B. D. Tait , C. Süsal , H. M. Gebel , et al., “Consensus Guidelines on the Testing and Clinical Management Issues Associated With HLA and Non‐HLA Antibodies in Transplantation,” Transplantation 95, no. 1 (2013): 19–47, 10.1097/TP.0b013e31827a19cc. 23238534

[2] P. Loiseau , K. Amokhrane , J. Visentin , V. D. Kheav , S. Caillat‐Zucman , and J. L. Taupin , “Use of Single‐Antigen Flow Beads Assays to Assess Anti‐HLA Donor‐Specific Antibody Strength,” Biology of Blood and Marrow Transplantation 22, no. 2 (2016): 394–395, 10.1016/j.bbmt.2015.11.006. 26597077

[3] G. A. Böhmig and L. Rostaing , “IdeS to Desensitize Organ Allograft Recipients,” Nature Reviews. Nephrology 13, no. 11 (2017): 666–668, 10.1038/nrneph.2017.128. 28890539

[4] E. Huang and S. C. Jordan , “Immunoglobulin G‐Degrading Enzyme of *Streptococcus pyogenes* (IdeS), Desensitization, and the Kidney Allocation System: Complementary Approaches to Increase Transplantation in Highly HLA Sensitized Patients,” Clinical Journal of the American Society of Nephrology 13, no. 5 (2018): 799–801, 10.2215/CJN.12031017. 29523676 PMC5969484

[5] S. C. Jordan , T. Lorant , J. Choi , et al., “IgG Endopeptidase in Highly Sensitized Patients Undergoing Transplantation,” New England Journal of Medicine 377, no. 5 (2017): 442–453, 10.1056/NEJMoa1612567. 28767349

[6] T. Lorant , M. Bengtsson , T. Eich , et al., “Safety, Immunogenicity, Pharmacokinetics, and Efficacy of Degradation of Anti‐HLA Antibodies by IdeS (Imlifidase) in Chronic Kidney Disease Patients,” American Journal of Transplantation 18, no. 11 (2018): 2752–2762, 10.1111/ajt.14733. 29561066 PMC6221156

[7] R. Bockermann , S. Järnum , A. Runström , et al., “Imlifidase‐Generated Single‐Cleaved IgG: Implications for Transplantation,” Transplantation 106, no. 7 (2022): 1485–1496, 10.1097/TP.0000000000004031. 34966107 PMC9213077

[8] S. Ge , M. Chu , J. Choi , et al., “Imlifidase Inhibits HLA Antibody‐Mediated NK Cell Activation and Antibody‐Dependent Cell‐Mediated Cytotoxicity (ADCC) In Vitro,” Transplantation 104, no. 8 (2020): 1574–1579, 10.1097/TP.0000000000003023. 32732834

[9] E. Huang , A. Q. Maldonado , C. Kjellman , and S. C. Jordan , “Imlifidase for the Treatment of Anti‐HLA Antibody‐Mediated Processes in Kidney Transplantation,” American Journal of Transplantation 22, no. 3 (2022): 691–697, 10.1111/ajt.16828. 34467625 PMC9293130

[10] L. Couzi , P. Malvezzi , L. Amrouche , et al., “Imlifidase for Kidney Transplantation of Highly Sensitized Patients With a Positive Crossmatch: The French Consensus Guidelines,” Transplant International 36 (2023): 11244, 10.3389/ti.2023.11244. 37448448 PMC10336835

[11] J. Visentin , M. Vigata , S. Daburon , et al., “Deciphering Complement Interference in Anti‐Human Leukocyte Antigen Antibody Detection With Flow Beads Assays,” Transplantation 98, no. 6 (2014): 625–631, 10.1097/TP.0000000000000315. 25058376

[12] HLA Eplet Registry , accessed May 23, 2023, https://www.epregistry.com.br/.

[13] R. J. Brezski , M. Kinder , K. D. Grugan , et al., “A Monoclonal Antibody Against Hinge‐Cleaved IgG Restores Effector Function to Proteolytically‐Inactivated IgGs In Vitro and In Vivo,” MAbs 6, no. 5 (2014): 1265–1273, 10.4161/mabs.29825. 25517311 PMC4623506

[14] S. Béland , O. Désy , R. El Fekih , et al., “Expression of Class II Human Leukocyte Antigens on Human Endothelial Cells Shows High Interindividual and Intersubclass Heterogeneity,” Journal of the American Society of Nephrology 34, no. 5 (2023): 846–856, 10.1681/ASN.0000000000000095. 36758118 PMC10125628

[15] G. Hönger , N. Krähenbühl , S. Dimeloe , M. Stern , S. Schaub , and C. Hess , “Inter‐Individual Differences in HLA Expression Can Impact the CDC Crossmatch,” Tissue Antigens 85, no. 4 (2015): 260–266, 10.1111/tan.12537. 25786570

[16] A. R. Tambur , N. D. Herrera , K. M. K. Haarberg , et al., “Assessing Antibody Strength: Comparison of MFI, C1q, and Titer Information,” American Journal of Transplantation 15, no. 9 (2015): 2421–2430, 10.1111/ajt.13295. 25930984

