# Supplementary figures and images for: Biological Effects of F(ab′)2 Fragments Generated by Imlifidase From Anti‐HLA IgG Antibodies From Transplant Patients
Source: HLA. 2025 Dec 14;106(6):e70502. doi: 10.1111/tan.70502 (PMC12703217; doi:10.1111/tan.70502)

Supplementary Figure 1:

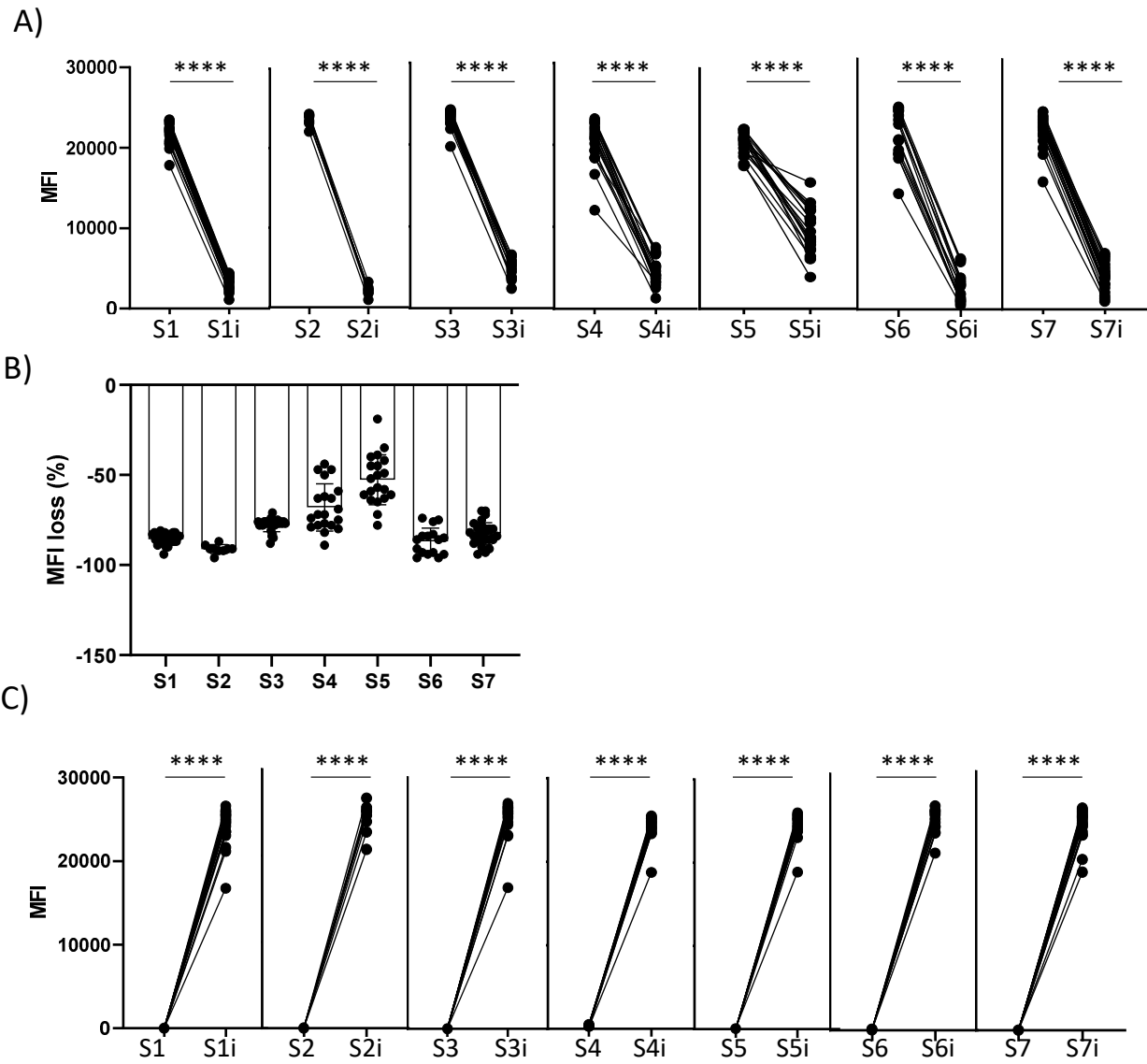

Supplement: Supplementary file 1 — Figure S1: IgG cleavage by imlifidase in the sera studied. Imlifidase‐treated sera S1i to S7i and non‐treated sera S1 to S7 were tested in LSA assay with an anti‐Fc (Panel A) or an anti‐Hinge conjugate (Panel C). Unpaired‐t‐tests were performed for each serum tested, presented in the graphs by asterisks as **** for p < 0.0001. Bar chart of percentages MFI loss in digested sera were depicted in panel B. [file TAN-106-e70502-s002.pdf]

Supplementary Figure 2:

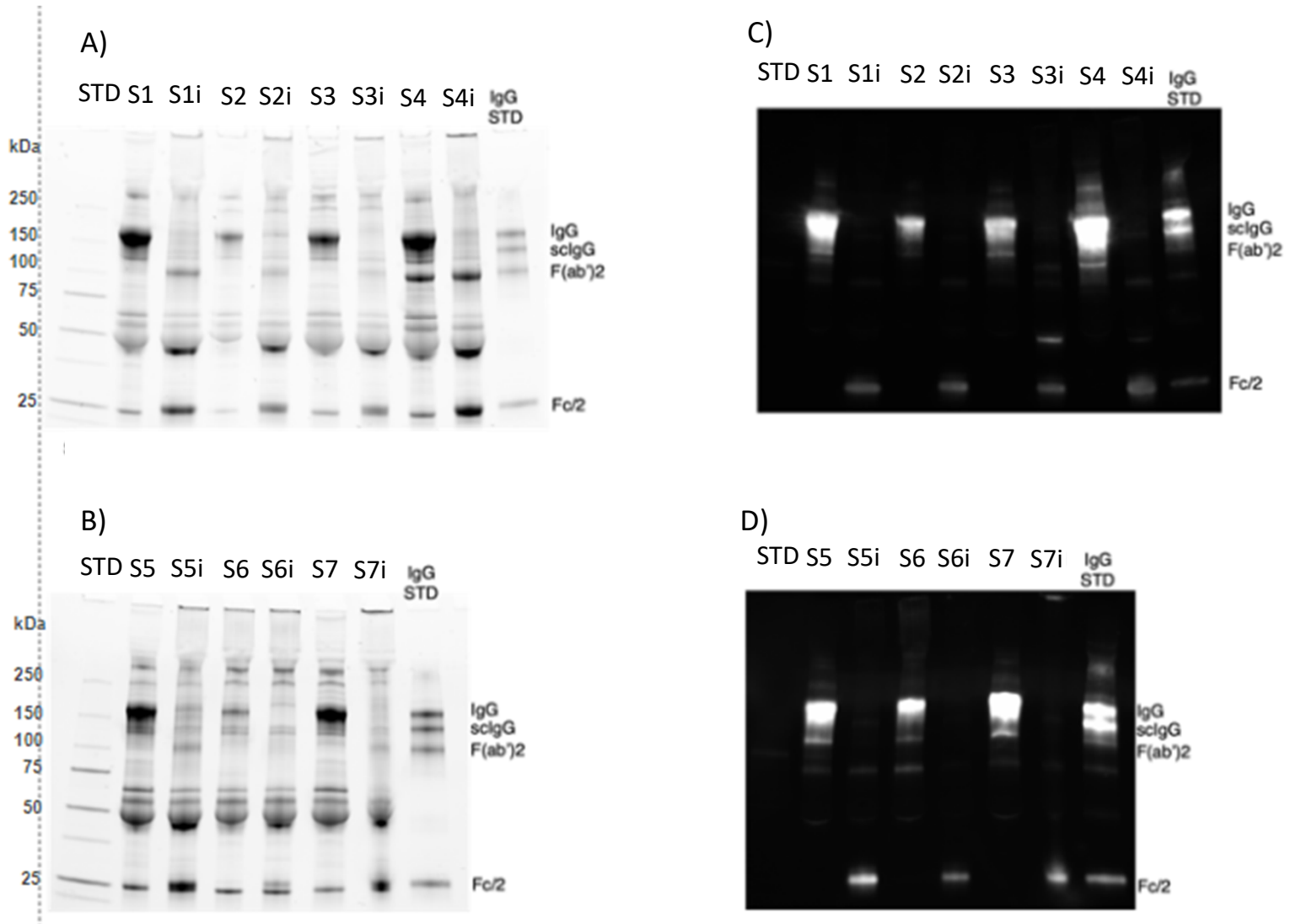

Supplement: Supplementary file 2 — Figure S2: SDS‐PAGE and Western Blot analysis of imlifidase cleavage. SDS‐PAGE (Panels A and B) and Fc‐specific Western Blot (Panels C and D) were performed on non‐treated sera S1 to S7 and imlifidase‐treated sera S1i to S7i to evaluate the efficacy of IgG cleavage by imlifidase. STD = molecular size stand. Experiment was performed as follows. For size separation, the serum samples were loaded onto 10‐well 4%–20% Mini PROTEAN TGX SDS‐PAGE (Biorad, Hercules, California) and run at 200 V for 40 min. Gels were activated for 2 min and picture acquired by ChemiDoc MP system (Biorad) using Image Lab5.2. For Fc‐specific Western Blot analysis, gels were shortly rinsed in water and blotted onto nitrocellulose using Mini Trans‐Blot Turbo Transfer Packs (#1704158, Biorad) 2.4 A on 25 V for 7 min. Membranes were blocked in 5% Skim milk powder for 60 min. Membranes were incubated with Biotin‐SP F(ab′)2 Fragment Goat Anti‐Human IgG Fcg‐specific (#109‐066‐098, Jackson ImmunoResearch, West Grove, Pennsylvania). Streptavidin‐AF647 diluted in Tris Buffered Saline‐Tween was used for detection on ChemiDoc MP (Biorad) with suitable filter. For S2i, S3i and S6i, serum sample preparations even under non‐reducing conditions, seems to show reducing activity in SDS‐PAGE assays, showing itself as 45 kDa Fab′ bands in the imlifidase cleaved samples. The 100 kDa band in the S4 sample is probably caused by a different serum protein than F(ab′)2. This might be due to treatment differences or disease status, for example, acute phase proteins in the serum. [file TAN-106-e70502-s001.pdf]
